# The potential causes of myasthenia and fasciculations in severely ill ME/CFS patients: the role of disturbed electrophysiology

**DOI:** 10.3389/fphys.2025.1693589

**Published:** 2026-02-02

**Authors:** Klaus J. Wirth, Jürgen M. Steinacker

**Affiliations:** 1 Mitodicure GmbH, Kriftel, Germany; 2 Institute of Rehabilitation Medicine Research at Ulm University, Ulm, Germany

**Keywords:** myalgic encephalomyelitis/chronic fatigue syndrome, long COVID, post-acute infection syndrome, myasthenia, loss of force, fasciculation, severely ill ME/CFS patient, Na^+^/K^+^-ATPase

## Abstract

Patients with severe myalgic encephalomyelitis/chronic fatigue syndrome (ME/CFS) are bedridden and suffer from hypersensitivities to light and noise, severe orthostatic intolerance reducing cerebral blood flow, and skeletal muscle symptoms, including loss of force, fatigue, pain, fasciculations, and cramps. Because neurological investigations exclude neuronal causes of myasthenia, we hypothesize a muscular pathomechanism. In previous articles, we considered insufficient activity of the Na^+^/K^+^-ATPase to be the main cause of mitochondrial damage via high intracellular sodium that reverses the transport mode of the sodium-calcium-exchanger to import calcium, causing calcium overload. Low Na^+^/K^+^-ATPase activity also causes sarcolemmal depolarization, leading to less effective action potential propagation and loss of force. Depolarization brings the membrane potential closer to the threshold potential, causing hyperexcitability that explains fasciculations and cramps. These increase sodium influx during excitation to further increase the workload of Na^+^/K^+^-ATPase. Thereby, depolarization causes further depolarization. Higher intracellular sodium favors calcium overload and mitochondrial damage, which lowers the energy supply of Na^+^/K^+^-ATPase and increases the reactive oxygen species, further inhibiting Na^+^/K^+^-ATPase. The muscle is in a state of depolarization even at rest. Depolarization and mitochondrial damage reinforce each other. Thus, dysfunction of Na^+^/K^+^-ATPase as a single mechanism can explain the different skeletal muscle symptoms of severely ill ME/CFS patients, comprising loss of force, fatigue, and fasciculations.

## 1 Introduction

Myalgic encephalomyelitis/chronic fatigue syndrome (ME/CFS) is a severe, frequent, and debilitating disease. There is an overlap with post-acute infection syndromes (PAISs), which demonstrate similar symptom profiles irrespective of the infectious agent (Choutka et al., 2022; Kedor et al., 2022; Peter et al., 2025). The prevalence of ME/CFS and PAIS has increased significantly because of SARS-CoV-2 infections. During the first wave of the COVID-19 pandemic, the incidence of developing PAIS was 6.5%–15% of those infected (Hill et al., 2022). PAIS after SARS-CoV-2 infection contributes substantially to the cumulative health burden in populations (Choutka et al., 2022; Kedor et al., 2022; Peter et al., 2025; Bowe et al., 2023; Steinacker and Klinkisch, 2025). ME/CFS presents with a plethora of symptoms, including severe fatigue, cognitive dysfunction, and exercise intolerance with post-exertional malaise (PEM), which are exacerbated by physical, emotional, or cognitive stress. Chronic whole-body pain, joint and muscle aches, and dyspnea have also been reported (Peter et al., 2025; Carruthers et al., 2011; Ballouz et al., 2023). Orthostatic intolerance, tachycardia, and palpitations are prevalent. An inability to stand upright or even to sit for a long time is found in more severely ill ME/CFS patients, who are often housebound or bedridden (van Campen et al., 2020; van Campen et al., 2023). Neurocognitive symptoms include the inability to concentrate, read, or memorize, and a cognitive cloudiness described as “brain fog.” Hypersensitivities to light, noise, and smell are reported. Sleep disorders are common, along with gastrointestinal symptoms (Peter et al., 2025; Carruthers et al., 2011; Ballouz et al., 2023). Despite these partially debilitating symptoms and the increasing prevalence, medicine has failed to define a pathophysiological picture. Clinical neurological investigations have not found disturbances in nerve conduction velocity; neuroimaging does not reveal spinal or cerebral pathologies. There is limited knowledge of mechanisms. Mechanisms identified to date include inflammation, formation of reactive oxygen species (ROS), proinflammatory cytokines, and activation of the inflammasome, in addition to disturbances in T-cell function and the secretory immune system and B-cell function, followed by complement activation, immune thrombosis, and decreased tissue blood flow (Ryabkova et al., 2019; Cervia-Hasler et al., 2024; Haunhorst et al., 2024). This article focuses on muscle weakness as an important contribution to the burden of disease. The inability to perform even low-intensity endurance exercises, increasing weakness after a few muscle contractions, and the loss of muscle strength for single contractions characterize myasthenia in ME/CFS. Skeletal muscle incapacity and myasthenia are important signs of disease severity (Carruthers et al., 2011; Conroy et al., 2021; Sommerfelt et al., 2023). Accordingly, handgrip strength inversely correlates with the severity of fatigue, disability, and symptoms (Paffrath et al., 2024; Jäkel et al., 2021; Nac et al., 2018; do Amaral et al., 2024). Muscle tremor or fasciculations have only been reported recently (Blitshteyn et al., 2024). In the Yale LISTEN trial, which included 423 subjects after COVID-19, 37% retrospectively reported “internal tremors, or buzzing/vibration.” Compared to other patients, participants with internal tremors reported worse health and had higher rates of new-onset mast cell disorders (11% vs. 2.6%) and neurological conditions (22% vs. 8.3%) (Zhou et al., 2024). As no neurological causes have been found, a muscular pathomechanism explaining loss of force, fasciculations (or tremor), cramps, and muscular pain should instead be assumed. The focus of this article is on the potential causes of disturbed muscle function and muscle symptomatology. In many other diseases, decisive pathophysiological insights have been gained from severe cases and have strongly contributed to our understanding of the pathophysiology of milder cases. Insights concerning the pathophysiology of ME/CFS, however, mainly come from patients with less severe disease because bedridden patients, unable to leave their homes, have not been accessible to medical research.

Cardiopulmonary exercise testing (CPET), which provides objective metabolic data in patients with mild to moderate symptomatology, demonstrated reduced maximal oxygen consumption (Appelman et al., 2024; Bizjak et al., 2024; Joseph et al., 2021), an early appearance of anaerobic metabolism during exercise, and deregulated energy metabolism (de Boer et al., 2022; Hoel et al., 2021).

In more than 50% of post-COVID and ME/CFS patients, mitochondria in skeletal muscles show damage to their internal structures (cristae) and shape, including lysis. In some cases, neighboring sarcomeres also show severe damage (Bizjak et al., 2024). Local and systemic metabolic disturbances, decreased oxidative phosphorylation, severe exercise-induced myopathy, and tissue infiltration of amyloid-containing deposits have been found (Appelman et al., 2024; Bizjak et al., 2024). However, these studies only involved patients who were able to exercise, which means that they had mild-to-moderate disease severity. Muscular force, measured as handgrip strength, is known to be decreased in ME/CFS and PAIS, and it has been shown to correlate with symptoms in patients who are still able to visit a medical unit (Paffrath et al., 2024; Nac et al., 2018; do Amaral et al., 2024).

## 2 Potential causes of myasthenia in ME/CFS

Four possible causes must be considered in the discussion on the loss of muscular force:

1. Lack of energy due to mitochondrial dysfunction.
2. Atrophy due to inactivity in the intention to avoid PEM and due to immobilization (deconditioning).
3. Skeletal muscle damage.
4. Electrophysiological causes that lead to insufficient excitation and recruitment of muscle fibers upon neuromuscular activation.

1) Skeletal muscle mitochondrial dysfunction can explain the loss of endurance and of force-endurance. However, a significant loss of force, even with the first muscular action after a sufficiently long rest, is not explained by a lack of energy due to mitochondrial dysfunction. This is because certain skeletal muscles are physiologically glycolytic, including the most vigorous muscles (which are made up of fast-twitch type 2b fibers). We have learned from SARS-CoV-2 infections that, even in asymptomatic athletes, VO_2_max is decreased after 3 months and much more so in those with symptoms (Vollrath et al., 2022). Viral proteins likely alter the mitochondrial epigenome through histone modifications or by modulating metabolite substrates, potentially silencing OXPHOS gene expression (Guarnieri et al., 2024).

2) Skeletal muscle atrophy and deconditioning as causes of muscle weakness have been excluded by a recent, careful investigation comparing healthy individuals after 6 weeks of bed rest with ME/CFS patients (Ch et al., 2025). Patients with long COVID and ME/CFS did not show muscle atrophy but did have fewer capillaries and more glycolytic fibers (Appelman et al., 2024; Ch et al., 2025; Scheibenbogen and Wirth, 2025). In contrast to healthy subjects after 6 weeks of bed rest, skeletal muscle in ME/CFS patients does not really rest, as patients suffer from cramps and fasciculations. A key assumption in our hypothesis is that calcium in skeletal muscle is too high and that calcium overload causes damage (Wirth and Scheibenbogen, 2021). Calcium pathways are indeed discussed as constituting the hypertrophic signal (Semsarian et al., 1999). It may oppose the tendency for atrophy by inactivity and thus prevent atrophy even in bedridden ME/CFS patients.

3) Although skeletal muscle damage has been shown in a recent study, this damage is most likely not extensive enough to cause a severe loss of force (Appelman et al., 2024). Skeletal muscle biopsies from severely ill patients are needed to clarify this issue.

4) In the section that follows, we argue that the main and common cause of both loss of force and fasciculations could be a disturbed electrophysiology of skeletal muscle. We try to explain how muscular symptoms, including loss of force and endurance, fasciculations, and pain, are causally related.

## 3 The potential role of the Na^+^/K^+^-ATPase in the physiology and pathophysiology of ME/CFS

A key assumption in our concept of a unifying disease hypothesis for ME/CFS, as published in previous articles, is that the Na^+^/K^+^-ATPase is dysfunctional (Wirth and Scheibenbogen, 2021; Wirth and Löhn, 2024). The causes are insufficient hormonal stimulation, active inhibition by ROS, and lack of ATP due to mitochondrial dysfunction. Ouabain, an inhibitor of the Na^+^/K^+^-ATPase, impressively demonstrates that inhibition of the Na^+^/K^+^-ATPase leads to a loss of muscle force upon electrical stimulation in isolated skeletal muscle and depolarization of the resting membrane potential (Clausen et al., 1993; Murphy and Clausen, 2007; Nielsen and Clausen, 1996; Wareham, 1978). Not only is sodium efflux decreased because of an insufficient Na^+^/K^+^-ATPase-activity, but sodium influx is also strongly increased by complex perfusion disturbances in PAIS, increasing the activity of the proton–sodium exchanger subtype 1 (NHE1), which is explained at length in a previous publication (Wirth and Löhn, 2024). This increases the workload of the already impaired Na^+^/K^+^-ATPase, leading to intracellular sodium loading, as shown in the skeletal muscles of ME/CFS patients (Petter et al., 2022). Sodium overload, in turn, causes calcium overload and associated calcium-induced damage at high intracellular sodium concentrations. The sodium–calcium exchanger (NCX) changes its transport direction to import calcium instead of exporting it at high intracellular sodium concentrations. The sodium concentration at which this occurs is referred to as the reverse mode threshold of the NCX. We see the reverse mode threshold of the NCX as the biological basis for the clinical PEM threshold (Wirth and Scheibenbogen, 2021). These pathomechanisms can also fully explain the observed skeletal muscle damage after exercise (Appelman et al., 2024).

## 4 The physiological role of the Na^+^/K^+^-ATPase in muscle electrophysiology and metabolism

Na^+^/K^+^-ATPase generates electrochemical ion gradients between the intracellular and extracellular spaces by exporting three Na^+^ ions against the cellular import of two K^+^ ions (Clausen, 2008; Pirkmajer and Chibalin, 2016). These gradients are responsible for the physiological resting membrane potential and the driving forces of excitation and propagation of action potentials. With diminished Na^+^/K^+^-ATPase activity, the resting membrane potential becomes more positive and gets closer to the threshold potential (depolarized). The consequence is hyperexcitability: thus, otherwise subthreshold stimuli can cause excitation, which is less efficacious, leading to reduced force development. Prolonged depolarization of the sarcolemma inactivates voltage-gated Na^+^ channels and impairs neuromuscular transmission. Na^+^/K^+^-ATPase accelerates repolarization and restores the excitability and contractility of skeletal muscle. Furthermore, by limiting K^+^ loss from contracting skeletal muscle, Na^+^/K^+^-ATPase activity also helps prevent or at least blunts exercise-induced extracellular interstitial hyperkalemia, which has depolarizing effects, increasing hyperexcitability. Na^+^/K^+^-ATPase activity also maintains the high intracellular K^+^ concentration that, together with chloride conductivity, forms the resting membrane potential in skeletal muscle.

During exercise, Na^+^/K^+^-ATPase is hormonally stimulated by protein kinase A (PKA) via cyclic adenosine monophosphate (cAMP), which is activated by ß2-adrenergic receptors and by calcitonin-gene-related peptide (CGRP) (Pirkmajer and Chibalin, 2016; Clausen, 2003; Nielsen and Harrison, 1998). At rest, Na^+^/K^+^-ATPase is stimulated by low concentrations of acetylcholine via the nicotinergic receptor and by insulin. Insulin stimulates Na^+^/K^+^-ATPase directly via protein kinase C and by its translocation from the cytoplasm to the cell membrane (Pirkmajer and Chibalin, 2016). A third indirect mechanism related to its energy supply will be explained below.

Na^+^/K^+^-ATPase is not only responsible for creating membrane excitability, but it is also involved in energetic processes. By maintaining low intracellular Na^+^ concentrations, Na^+^/K^+^-ATPase promotes Na^+^-coupled transport and uptake of substrates (e.g., carnitine, inorganic phosphate, and amino acids such as glutamine and alanine), which play key roles in the energy metabolism of skeletal muscle (Odoom et al., 1996; Zorzano et al., 2000). Furthermore, it provides the sodium gradient for proton export via the sodium–proton exchanger. Pyruvate kinase activity is dependent on the physiologically intracellular (high) potassium concentration (Page and Di Cera). Proper Na^+^/K^+^-ATPase activity keeps intracellular potassium high and thus prevents the decrease in pyruvate kinase activity. The latter is dependent on K^+^, thus preserving the energy supply via glycolysis even at normal oxygen pressure (aerobic glucose utilization) for its own energy supply. Hence, insufficient Na^+^/K^+^-ATPase activity can also have negative effects on skeletal muscle metabolism and not only impair electrophysiology.

## 5 Potential disturbances of Na^+^/K^+^-ATPase in ME/CFS

Autoantibodies against ß2-adrenergic receptors and the high tendency for desensitization of this receptor by the high sympathetic tone found in ME/CFS (for which orthostatic stress may be the most important cause) may cause dysfunction of the Na^+^/K^+^-ATPase (Scheibenbogen et al., 2018; Lohse et al., 2003). The G protein-coupled receptor (CGRP) is released from small nerve fibers; however, small fiber neuropathy, which is frequently reported in ME/CFS (Joseph et al., 2021; Abrams et al., 2022; Oaklander and Nolano, 2019), diminishes CGRP release and availability. Moreover, TRPM3 dysfunction is present in ME/CFS in leukocytes (Cabanas et al., 2019; Cabanas et al., 2018; Eaton-Fitch et al., 2022). TRPM3 is not only present in leukocytes but also expressed and involved in the release of neuropeptides from sensory nerves, so its dysfunction may reduce CGRP release even before overt small nerve fiber degeneration occurs (Alonso-Carbajo et al., 2019; Held et al., 2015; Held and Tóth, 2021). Mitochondrial dysfunction develops in fully developed ME/CFS, and the high energy need of the Na^+^/K^+^-ATPase limits its activity. ROS produced due to mitochondrial dysfunction can even inhibit the Na^+^/K^+^-ATPase through stimulation of glutathionylation (Pirkmajer and Chibalin, 2016; Clausen, 2003; Jammes et al., 2020).

Insulin resistance of skeletal muscle, likely of a mild degree, has been reported in ME/CFS patients (Al Masoo et al., 2025; Allain et al., 1997; Beentjes et al., 2025; Al-Hakeim et al., 2023; Hoel et al., 2021). Insulin stimulates Na^+^/K^+^-ATPase at rest. Due to insufficient stimulation, the ionic situation in skeletal muscle in patients with insulin resistance may not recover sufficiently from previous muscle work for the next activity. Patients may then start muscle activity under unfavorable intracellular ionic conditions, which lead to an early increase in intracellular sodium and calcium, explaining a low PEM threshold in our hypothesis (Pirkmajer and Chibalin, 2016; Clausen, 2003; Nielsen and Harrison, 1998).

## 6 High workload and energy consumption of the Na^+^/K^+^-ATPase by sarcolemmal depolarization and fasciculations

**FIGURE 1:**
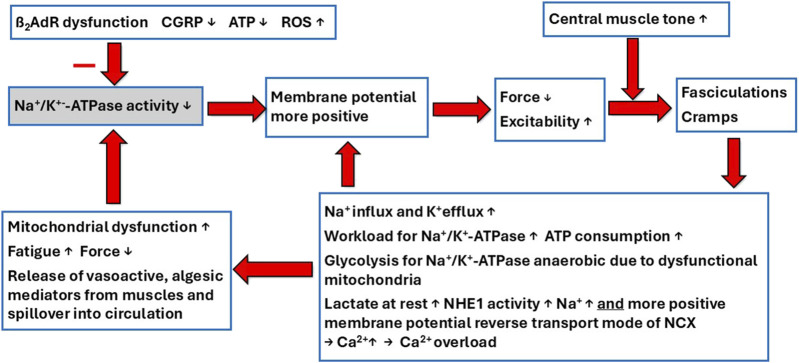
Presumed skeletal muscle pathophysiology of severely ill ME/CFS patients. Insufficient Na^+^/K^+^-ATPase activity due to dysfunction of its hormonal stimuli (ß2-adrenergic agonism and CGRP), ATP-deficiency, and active inhibition by ROS causes skeletal muscle membrane depolarization. This impairs muscle force and increases muscle excitability and sensitivity to central stress-induced increases in muscle tone. Depolarization and increased central muscle tone cause inadequate excitations, such as fasciculations and cramps, leading to sodium influx and potassium efflux, resulting in a higher workload for the already insufficient Na^+^/K^+^-ATPase. The latter becomes even more incapacitated in its ability to restore a stable physiological resting membrane potential. Thus, depolarization causes further depolarization in a vicious cycle. This increases energy consumption, lactate production, and intracellular sodium, and, finally, intracellular calcium to further damage the mitochondria by calcium overload. These disturbances are already present at rest or are caused by minimal efforts.

For reasons that will be explained below, central muscle tone is probably also increased to favor spontaneous, uncoordinated excitations. Sarcolemmal depolarization brings the membrane at rest closer to the action potential threshold. As a result, skeletal muscle is sensitized to increases in central muscle tone and centrally induced excitations. Uncontrolled excitations caused in this way become manifest as muscle fasciculations and cramps and clearly indicate hyperexcitability. It cannot be excluded that there are frequent, unnoticed excitations of single muscle fibers. Alterations in EMG activity have indeed been reported in ME/CFS and long COVID in less severe cases (Agergaard et al., 2023; Jammes et al., 2020). Although these M-wave alterations are rather nonspecific and therefore do not reveal a particular cause, M-wave alterations have been previously attributed to a dysfunctional Na^+^/K^+^-ATPase inhibited by ROS (Jammes et al., 2020; Jammes and Retornaz, 2019). Excitations cause sodium influx during depolarization and potassium efflux during repolarization, strongly increasing the workload of the Na^+^/K^+^-ATPase, which is already insufficient to restore the physiological action potential. Thus, the workload for the Na^+^/K^+^-ATPase also increases ATP consumption. It is estimated that 5%–10% of oxygen consumption in skeletal muscle at rest is coupled to Na^+^/K^+^-ATPase activity (Pirkmajer and Chibalin, 2016). In the event of frequent excitations, the workload and energy consumption of the Na^+^/K^+^-ATPase strongly increase, even at rest. Muscle contractions are the strongest stimulus for Na^+^/K^+^-ATPase activation. In healthy muscles, direct stimulation of isolated skeletal muscle rapidly increases Na^+^ efflux 20-fold (Nielsen and Harrison, 1998). It is self-explanatory that an impaired Na^+^/K^+^-ATPase function, which we strongly assume is present in ME/CFS, cannot increase its activity by a factor of 20 during exercise. This explains the electrophysiological loss of force, exercise intolerance, and other pathomechanisms. The latter includes intracellular sodium loading, leading to subsequent calcium overload via the reverse mode of the NCX. Additionally, the reverse mode of the NCX is favored by a more positive membrane potential (Blaustein and Lederer, 1999). Figure 1 shows the presumed sequence of skeletal pathomechanisms causing Na^+^/K^+^-ATPase dysfunction and its consequences for skeletal muscle pathophysiology in ME/CFS.

## 7 Energy supply of the Na^+^/K^+^-ATPase and its disturbances

The ATP supply of the Na^+^/K^+^-ATPase in skeletal muscle strongly depends on glycolysis (Dutka and Lamb, 2007). In aerobic muscle fibers, in contrast to glycolytic muscle fibers, it is very likely that the pyruvate generated by glycolysis for the Na^+^/K^+^-ATPase’s ATP supply is used up in the citrate cycle after decarboxylation to acetyl-CoA by the nearby mitochondria, the subsarcolemmal mitochondria, for the generation of the contractile force and work. In ME/CFS, however, these subsarcolemmal mitochondria show signs of damage, and therefore, it is likely that glucose metabolism becomes anaerobic to produce more lactate, even in aerobic muscle fibers. Lactate production increases proton concentrations, which are mainly expelled by the sodium–proton exchanger NHE1, further increasing intracellular sodium levels and the workload for the impaired Na^+^/K^+^-ATPase, thereby raising the likelihood of calcium overload. This depolarized state causes frequent, inadequate excitations with sodium influx and potassium efflux for repolarization, but the impaired Na^+^/K^+^-ATPase can no longer restore a stable resting membrane potential, which would put an end to the permanent excitations. The skeletal muscle is thus caught in the “depolarization trap.” Therefore, sarcolemmal depolarization generates further depolarization. This raises the workload and energy consumption of Na+/K+−ATPase, but the energy supply is limited due to ineffective glucose metabolism and mitochondrial dysfunction.

Physiologically, excitation and skeletal muscle work (i.e., contraction) are coupled. Therefore, the energetic processes for electrophysiological activity and physical work should also be physiologically coupled. Excitations due to a depolarized state of the sarcolemma and increased excitability, as assumed here, lead to a high workload of the Na^+^/K^+^-ATPase already at rest, dissociating the (high) electrophysiological energetic demand from the demand for physical work for contraction (no demand at rest). Pyruvate would then not be utilized by the citrate cycle, and therefore, lactate would be generated because pyruvate is in equilibrium with lactate. This may explain why some patients show elevated lactate levels at rest (Ghali et al., 2019). This would impair glycolysis and the energy supply of the Na^+^/K^+^-ATPase, thereby weakening its activity. This does not necessarily mean that there is a positive correlation between a lactate increase at rest and skeletal muscle depolarization and muscular symptoms. In resting conditions, enhanced anaerobic glycolysis can still effectively provide enough energy for the increased needs of the Na^+^/K^+^-ATPase via anaerobic glycolysis to mitigate or prevent depolarization. However, insulin resistance is present, reducing glucose uptake in skeletal muscle, as referenced above, and even impairing anaerobic glycolysis as the energy source for the Na^+^/K^+^-ATPase and thereby its function, favoring depolarization and symptoms (Hoel et al., 2021; Pirkmajer and Chibalin, 2016; Al Masoo et al., 2025; Allain et al., 1997; Beentjes et al., 2025; Al-Hakeim et al., 2023). Insulin resistance results in lower cellular glucose and pyruvate levels, which could explain why lactate levels remain (paradoxically) low and, second, when complex I dysfunction is present, why the import of pyruvate into the respiratory chain as acetyl-CoA is further diminished.

Overall, insulin is important in the activity of the Na^+^/K^+^-ATPase via several mechanisms: providing glucose for its energy supply, stimulating its activity via protein kinase C, and promoting its translocation from the cytoplasm to the cell membrane, as explained above.

During exercise, Na^+^/K^+^-ATPase activity increases, although probably less in patients with ME/CFS than in healthy controls, contributing to exercise intolerance. Pyruvate increases from the glycolytic pathway needed for the ATP supply to the Na^+^/K^+^-ATPase. The limiting factor for pyruvate utilization in aerobic muscles is their low oxidative capacity due to skeletal muscle mitochondrial dysfunction. This can explain the increased lactate levels during exercise. Thus, the increases in lactate at rest and during exercise may have different causes in ME/CFS patients.

## 8 Central muscle tone and depolarized sarcolemma interact to cause inappropriate excitations

We have mentioned that the central muscle tone is elevated. Three potential causes for an increased muscle tone that favors spontaneous skeletal muscle excitations of the depolarized muscle membrane must be considered based on new recognitions and findings.

1. TRPM3 dysfunction.
2. Autoantibodies against serine/arginine repetitive matrix protein 3 (SRRM3).
3. Autoantibodies against the alpha2C-adrenergic receptor (alpha2C-R).

1) TRPM3 dysfunction is present in many ME/CFS patients (Cabanas et al., 2019; Cabanas et al., 2018; Eaton-Fitch et al., 2022). As explained in a previous article, TRPM3 appears to be involved in the GABA system. Its dysfunction could lower the release of GABA and thus increase muscle tone (Held and Tóth, 2021; Löhn and Wirth, 2024; Seljeset et al., 2023).

2) Autoantibodies have been reported against SRRM3 (Hoheisel et al., 2024). SRRM3, which is expressed in the brain, is a regulator of alternative splicing (essential for motor coordination) and contributes to the switch of GABA-ergic signaling from excitatory to inhibitory (Nakano et al., 2019). Remarkably, a single nucleotide variant in SRRM3 was found to be associated with ME/CFS, strengthening the role of this protein and GABA in ME/CFS pathophysiology (Schlauch et al., 2016).

3) Autoantibodies have been reported against alpha2C-R. Alpha2C-R could be involved in ME/CFS, as already suggested in a previous article, simply based on its physiological functions and its tendency to desensitize to catecholamines (Artalejo and Olivos-Oré, 2018; Wirth et al., 2021). Its dysfunction could explain some symptoms of ME/CFS beyond the effect on skeletal muscle tone, which will be explained below. Severe sleep disturbance, likely linked to hypervigilance and high sympathetic tone, is a hallmark of ME/CSF (Nelson et al., 2019; Wyll et al., 2009). Recent findings even show autoantibodies against this important receptor, further strengthening the previous assumption that this receptor could be relevant and could be dysfunctional in ME/CFS (Hoheisel et al., 2024). As a postsynaptic receptor, alpha2C-R is present on veins with a vasoconstrictor function (Gyires et al., 2009). Inhibition by autoantibodies would cause orthostatic dysfunction due to insufficient venous contraction, reducing cardiac preload, which would increase sympathetic tone for compensation (orthostatic stress). Alpha2C-R is also a presynaptic receptor on sympathetic nerve endings, acting as an autoreceptor to limit and modulate catecholamine release (Gyires et al., 2009). Its dysfunction would cause excessive norepinephrine release and potentially cause vasoconstriction during sympathetic activation. Norepinephrine released excessively by the synergistic effects of the two mechanisms explained above would have overshooting vasoconstrictor effects because the vasodilatory effect of the intact endothelium, which physiologically opposes vasoconstriction, is also dysfunctional (Scherbakov et al., 2020). Thus, vasospasms in the periphery (Raynaud symptoms) and orthostatic cerebral vasoconstriction could be explained satisfactorily (van Campen and Visser, 2025). In the brain, alpha2C-R is expressed in activating noradrenergic brain regions such as the locus coeruleus (Gyires et al., 2009; Rosin et al., 1993). Pharmacological agonists at this presynaptic alpha2C-adrenergic receptor cause sedation, muscle relaxation, and a mild analgesic effect. This enables us to understand the (opposite) effects that blocking autoantibodies could instigate by causing alpha2C-R dysfunction (Hayashi and Maze, 1993). Its dysfunction might increase pain perception, muscle tone, vigilance, and arousal, all of which are involved in the severe sleep disturbances and sympathetic hyperactivity typically found in ME/CFS (Nelson et al., 2019; Wyll et al., 2009; Wyller et al., 2008).

The role of the histamine system in skeletal muscle pathophysiology and tremor in ME/CFS is not fully understood. Recent findings have revealed a small mast cell population in skeletal muscle that is likely responsible for histamine secretion during exercise. These cells also target myeloid and vascular cells rather than myofibers in a paracrine manner (Van der Stede et al., 2025). High mast cell activation is more frequent in cases of muscle dysfunction and tremor in ME/CFS, suggesting a potential role (Zhou et al., 2024). However, the pathomechanisms by which histamine could disturb skeletal muscle function and cause tremor are not yet clear.

In this section, we have collected evidence that central skeletal muscle tone is increased by disturbances of the noradrenergic and the GABA neurotransmitter systems, leading to a predominance of excitatory over inhibitory neurotransmitters for skeletal muscle tone. In the presence of a depolarized skeletal muscle membrane, which induces peripheral hyperexcitability, the central increase in skeletal muscle tone facilitates the generation of irregular action potentials. These become clinically manifest as fasciculations, cramps, and tremors.

## 9 Skeletal muscle depolarization in severely ill patients is superimposed on mitochondrial dysfunction: a step model to explain the condition of severely ill ME/CFS patients

Overall, the muscles of severely ill ME/CFS patients may be caught in a state of depolarization and mitochondrial damage, even at rest. Even the slightest effort aggravates the condition. Energy-depleted muscles compensatorily release vasoactive mediators, such as bradykinin, prostaglandins, prostacyclin, and adenosine, to increase local blood flow and remove the energetic deficit (Wirth and Scheibenbogen, 2020); they also release histamine (Van der Stede et al., 2025). When excessively produced, these otherwise very labile mediators can reach every organ (spillover into systemic circulation). There, they can cause symptoms typical for ME/CFS such as edema, pain, and spasms due to their vasoactive, inflammatory, vascular leakage-inducing, algesic, hyperalgesic, and spasmogenic effects (Wirth and Scheibenbogen, 2020). In skeletal muscle itself, these algesic mediators can cause muscular pain. In the kidney, mediators such as bradykinin and prostacyclin cause hyperexcretion of sodium and water by increasing renal blood flow and inhibiting distal tubular sodium reabsorption. These effects prevent an increase in renin to correct for the resulting hypovolemia (explaining the renin paradox). This renal mechanism, causing hypovolemia and increased vascular leakage, could play a significant role in orthostatic dysfunction and orthostatic stress (Wirth and Scheibenbogen, 2020).

Concerning skeletal muscle pain in ME/CFS, several mechanisms could be involved, including the release of algesic mediators such as bradykinin, skeletal muscle damage after exercise, cramps, and central sensitization, as explained above.

Our unifying hypothesis on the causes of the symptoms and pathophysiology of ME/CFS can explain the condition of severely ill patients without making new assumptions. Mitochondrial damage and skeletal muscle depolarization reinforce each other. They have the same cause: a dysfunction of ion transport, mainly due to a Na^+^/K^+^-ATPase dysfunction. Different pathomechanisms, including increased sodium influx into skeletal muscle, central pathomechanisms, risk factors, and triggers, contribute to these disturbances. It is the stringent application of the current concept that provides a single pathomechanism to explain loss of force, fatigue, and fasciculations of skeletal muscle.

**FIGURE 2:**
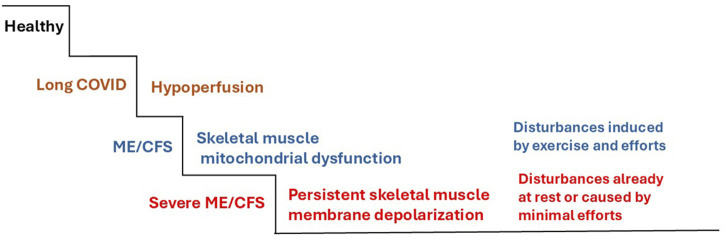
Step model that explains the condition of severely ill ME/CFS patients. Post-COVID-19 syndrome is mainly due to severe vascular disturbances due to an interaction between capillary microvascular and precapillary cardiovascular disturbances triggered by the virus itself or by excessive cytokine and immune responses, leading to hypoperfusion. In susceptible patients, skeletal muscle mitochondrial dysfunction develops to trigger ME/CFS as a self-maintaining vicious cycle in which ionic disturbances caused by insufficient Na^+^/K^+^-ATPase activity play a key role. In severely ill patients, further impaired Na^+^/K^+^-ATPase activity cannot maintain or restore the physiological resting membrane potential in skeletal muscle. The resulting membrane depolarization leads to loss of force, hyperexcitability, and further aggravation of mitochondrial dysfunction due to ionic disturbances, as shown in Figure 1.

Figure 2 shows a step model of ME/CFS arising from long COVID. During the long COVID stage, hypoperfusion is present, mainly caused by microvascular dysfunction (Kruger et al., 2024). From these perfusion disturbances, in the presence of risk factors, ME/CFS develops via skeletal muscle mitochondrial dysfunction. PEM, as an aggravation of the complaints, occurs with exercise and effort. The third stage involves severely ill patients with persisting membrane depolarization that develops because of insufficient Na^+^/K^+^-ATPase activity and mitochondrial dysfunction. These disturbances are persistent or induced by minimal effort.

**FIGURE 3:**
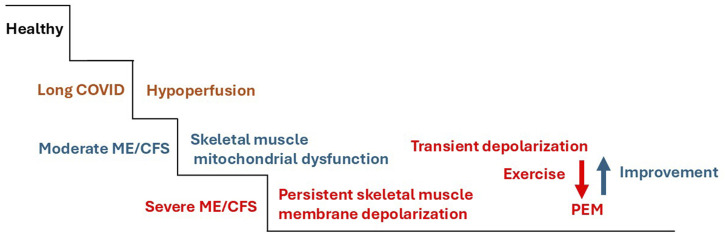
Reversible skeletal muscle membrane depolarization during severe episodes of PEM in patients with moderate ME/CFS. While skeletal muscle membrane depolarization is persistent in severely ill patients, in patients with moderate ME/CFS, muscle membrane depolarization may only be transiently present during longer-lasting or more severe episodes of PEM after excessive exertion. The patients, however, may be capable of restoring a stable physiological membrane potential, at least at rest, and maintaining it during moderate levels of exertion that do not cause PEM.

Based on these considerations, one can conclude that the skeletal muscles of patients with moderate disease that is in a prolonged episode of PEM could also be in a (transient) depolarized state. Skeletal muscles may be in a depolarized state for extended periods, but patients may be able to overcome excessive depolarization to return to the stage of mitochondrial dysfunction (Figure 3). In support of this idea, EMG changes were observed in approximately half of ME/CFS patients after a cycling exercise. The M-wave amplitude significantly decreased in leg muscles, and the M-wave duration significantly increased (Retornaz et al., 2023). This measurement with surface electrodes does not assess membrane voltage, but a surface summation potential, so it does not directly reveal sarcolemmal depolarization. The decrease in amplitude and the longer duration suggest a less effective recruitment of muscle fibers and a delayed propagation of the excitation along the skeletal muscle fibers that, after exclusion of neuronal causes, can be explained by membrane depolarization, as a fraction of excitatory sodium channels would have been in the inactivated state. This suggests that sarcolemmal depolarization can occur even in mild-to-moderate ME/CFS. Interestingly, in the group of patients developing exercise-induced M-wave alterations, resting values of handgrip strength were significantly lower, and symptoms were more serious than in patients without M-wave abnormalities. Hence, the known correlation between loss of muscle force and symptoms also seems to apply to EMG changes (disturbed excitability). This is not surprising, as we see the disturbed electrophysiology behind the demonstrated EMG changes as the cause for the loss of force.

These considerations also help explain why a loss of force after diagnosis correlates with symptoms and poor prognosis (Paffrath et al., 2024). The main cause for the loss of force, an insufficient Na^+^/K^+^-ATPase activity, is also the main cause for sodium-induced calcium overload that causes skeletal muscle mitochondrial dysfunction, the key pathomechanism in our ME/CFS disease concept.

## 10 Conclusion

Insufficient Na^+^/K^+^-ATPase activity can explain mitochondrial dysfunction via sodium-induced calcium overload, causing diminished oxidative phosphorylation in skeletal muscle, leading to a lack of energy, fatigue, and loss of endurance, along with a disturbance of skeletal muscle electrophysiology. The latter leads to chronic depolarization of the sarcolemma, which explains both loss of force due to impaired action potential propagation and fiber recruitment and fasciculations (hyperexcitability). Skeletal muscle depolarization may play a strong role in the myasthenia of these severely ill ME/CFS patients. It may also contribute to a vicious circle that increases sodium loading through inappropriate excitations, further increasing mitochondrial damage through calcium overload.

## Data Availability

The original contributions presented in the study are included in the article/supplementary material; further inquiries can be directed to the corresponding author.
